# Acute Exercise-Associated Metabolomic Remodeling and Associations with Oxidative State in Human Skeletal Muscle

**DOI:** 10.3390/metabo16100763

**Published:** 2026-10-10

**Authors:** Chang Liu, Qisen Han, Yao Yan, Biaoxu Tao, Meiling Liu, Xincheng Dai, Zhiqin Xu, Wei Jiang, Kai Zhao, Song Yan

**Affiliations:** 1 School of Sport Science, Beijing Sport University, Beijing 100084, China; c.liu@bsu.edu.cn (C.L.); 2024012768@bsu.edu.cn (Q.H.); taobx020907@gmail.com (B.T.); 2024210297@bsu.edu.cn (Z.X.); 2 China Basketball College, Beijing Sport University, Beijing 100084, China; 2024210467@bsu.edu.cn; 3 School of International Education and Exchange, Beijing Sport University, Beijing 100084, China; 2025011325@bsu.edu.cn; 4 School of Art, Beijing Sport University, Beijing 100084, China; 2023014432@bsu.edu.cn; 5 China Volleyball Academy, Beijing Sport University, Beijing 100084, China; 2430@bsu.edu.cn (W.J.); 2326@bsu.edu.cn (K.Z.); 6 School of Education, Beijing Sport University, Beijing 100084, China

**Keywords:** acute exercise, skeletal muscle, metabolomics, oxidative state, carnitine, NAD+, redox biology, machine learning

## Abstract

**Background:** Acute exercise rapidly reorganizes skeletal-muscle metabolism. The metabolite pools that remain responsive with aging, and their relationship to baseline oxidative state, remain incompletely defined. **Methods:** We analyzed 135 metabolites measured in 93 skeletal-muscle biopsies from 47 participants and matched baseline transcriptomic measurements. We tested paired post–pre changes, biochemical-class enrichment, baseline associations with a four-pathway oxidative transcript-state score, and participant-held-out prediction of acetylcarnitine response. False-discovery-rate control was applied within each analysis family. Bootstrap confidence intervals and a baseline-adjusted sensitivity analysis quantified predictive performance. **Results:** In exercise-trained older adults, 17 metabolites changed after acute exercise after FDR correction. Acetylcarnitine increased, whereas free carnitine, malate, ATP, and choline decreased. No exercise-trained-versus-normally active response contrast survived correction. The CoA–glutathione-annotated feature and NAD^+^ remained associated with the oxidative transcript-state score after adjustment for group and sex (partial ρ=0.512 and 0.514; both q=0.042). A four-metabolite TabPFN model predicted acetylcarnitine change with an out-of-fold $R^{2}$ of 0.412 (95% CI, 0.079–0.641), similar to the reference model ($R^{2}=0.399$, 95% CI, 0.054–0.625). Baseline acetylcarnitine alone achieved an AUC of 0.817 (95% CI, 0.680–0.941) for a twofold-response endpoint. Additional metabolites provided only modest incremental information after baseline adjustment. **Conclusions:** Acute exercise induced heterogeneous, distributed remodeling of skeletal-muscle metabolite pools. Baseline redox-related molecular state showed cross-omic coherence and contained information about subsequent acetylcarnitine response. Transcriptomic augmentation and more complex predictive architectures provided limited incremental benefit.

## 1. Introduction

Skeletal muscle rapidly reorganizes ATP turnover, substrate use, mitochondrial metabolism, lipid handling, and redox balance when exercise increases energetic demand. Human multi-omic studies indicate that metabolite and signaling changes can precede transcriptional adaptation, and that responses differ by exercise modality [1,2,3,4].

The magnitude and composition of this response vary with age, habitual training, muscle composition, and baseline metabolic context [5,6,7]. Bulk muscle profiles also combine myofiber, endothelial, stromal, and immune signals [8,9]. Redox-related metabolites, including NAD^+^, NADP^+^, glutathione-related compounds, coenzyme A intermediates, and organic acids, therefore provide plausible descriptors of the molecular state from which an acute response begins. These measurements remain pool-size indicators rather than direct measures of flux or mitochondrial function [10,11,12,13,14,15,16,17].

Important questions remain in human skeletal muscle. Which metabolite pools change reproducibly after an acute exercise challenge, and are the changes concentrated in one biochemical class or distributed across connected pathways? Are baseline metabolite levels associated with an independently derived oxidative transcriptional state? Does baseline molecular information predict individual response magnitude, and does transcriptomic information add beyond baseline metabolite state? These are separate estimands: cross-sectional association and response prediction should not be interpreted as causal treatment effects [18,19].

We reanalyzed complementary public datasets to address these questions. The primary analysis quantified within-person acute metabolomic remodeling. A secondary analysis tested baseline associations with a prespecified oxidative transcript-state score. Exploratory participant-held-out models evaluated acute acetylcarnitine change while retaining simple baseline comparators. We hypothesized that acute exercise would produce coordinated but heterogeneous metabolomic changes, that selected redox-related metabolites would covary with oxidative transcript state, and that baseline metabolites would provide more predictive information than transcriptomic augmentation.

## 2. Materials and Methods

### 2.1. Study Design and Data Sources

This was a secondary analysis of publicly available, de-identified human molecular datasets. The principal acute-exercise analysis used MTBLS12824 and the linked skeletal-muscle transcriptomic resource GSE330697 from the same source cohort. MTBLS12824 contained 135 annotated metabolites from 93 vastus lateralis biopsies obtained from 47 participants: 11 young, 15 normally active older, 16 exercise-trained older, and 5 physically impaired older participants. Complete paired metabolomics were available for 46 participants (11, 14, 16, and 5, respectively). Matched baseline metabolomic and transcriptomic measurements were available for 45 participants, and 43 participants had complete features for exploratory acetylcarnitine prediction. The source-study framing is consistent with evidence that exercise responses can remain measurable across insulin-resistant and aging phenotypes, although phenotype and response are not interchangeable estimands [20,21].

Participants completed a 60 min cycling bout after an overnight fast at approximately 50% of individual maximal workload and a cadence of approximately 60–70 rpm. Vastus lateralis biopsies were obtained before exercise and immediately after exercise. Additional public datasets (GSE158850, GSE159809, and GSE244120) were analyzed independently as contextual transcriptomic analyses because their tissues, interventions, platforms, and sampling schedules differed from the acute cohort. They were not treated as independent validation of the metabolomic response. The datasets and their analytical roles are summarized in Table 1.

### 2.2. Metabolomic Processing and Acute Response

The source metabolomics used high-resolution liquid chromatography–mass spectrometry with positive- and negative-ionization modes. We analyzed the deposited log_2_ relative-abundance matrix for 135 annotated metabolites. Metabolite identities were retained according to the annotations provided in MTBLS12824. The present secondary analysis did not independently re-identify metabolites from raw mass spectra. The CoA–glutathione feature was therefore analyzed using the deposited annotation supplied by the original metabolomic dataset. Participant identifiers, group assignments, and biopsy timing were reconciled using repository metadata and source manifests. For participant *i* and metabolite *j*, the acute response was defined as $\Delta M_{ij}=M_{ij,\mathrm{post}}-M_{ij,\mathrm{pre}}$. Positive values indicate an exercise-associated increase. Metabolite processing and annotation were retained at the deposited level, consistent with current metabolomics analysis workflows [22,23].

Group-specific paired tests were performed across the 135 metabolites. Benjamini–Hochberg correction was applied within each group-specific response family [24]. Direct contrasts of post–pre changes between exercise-trained and normally active older adults were corrected across the same 135 features. Confidence intervals were calculated for key effects, and participant-level sensitivity analyses preserved the paired structure. The deposited 2-hydroxyglutarate feature was not resolved into D- and L-enantiomers.

### 2.3. Biochemical Classes and Enrichment

Metabolites were assigned before testing to prespecified muscle-relevant classes: carnitine shuttle, NAD/redox, TCA, glycolysis/carbohydrate, amino-acid, nucleotide/energy, and choline/phospholipid metabolism. Overlapping membership was allowed when a metabolite had more than one established biochemical role. Pathway definitions were checked against KEGG, MSigDB, and gene-set knowledge resources [25,26,27]. One-sided Fisher exact tests assessed whether FDR-supported trained-older metabolites were overrepresented in each class; correction was applied across the declared classes. These tests were interpreted as biochemical organization of measured pools, not evidence of pathway flux.

### 2.4. Oxidative Transcript-State Score and Cross-Omic Analysis

For the linked skeletal-muscle transcriptomic dataset, genes with more than two counts in at least four samples were retained. Library sizes were normalized using the trimmed mean of M-values method, followed by precision-weighted log_2_ counts-per-million transformation using the edgeR–limma/voom workflow. Gene identifiers were mapped to current HGNC gene symbols before pathway-level analyses.

Four oxidative-metabolism pathways were prespecified from the Molecular Signatures Database (MSigDB, version 2025.1.Hs): oxidative phosphorylation, citrate cycle/tricarboxylic acid cycle, fatty-acid metabolism, and valine, leucine and isoleucine degradation. Only genes represented in both the corresponding gene set and the expression matrix were retained.

For each sample, normalized gene-expression values were ranked and converted to fractional ranks ranging from 0 to 100. A pathway score was calculated as the arithmetic mean of the fractional ranks of all available member genes. The oxidative transcript-state score was then defined as the unweighted mean of the four pathway scores. GSVA was not used to generate the primary oxidative-state score. The resulting score was interpreted as a relative transcriptomic representation of oxidative-metabolic organization rather than a direct measurement of mitochondrial respiration, ATP production, oxygen consumption, or substrate flux.

Because rank-based pathway scores depend on the expressed-gene universe, assay platform, and preprocessing procedure, absolute score values were considered dataset-specific. Consequently, oxidative-state scores were compared statistically only within individual datasets rather than directly across cohorts. Differences in absolute score distributions between datasets therefore do not imply proportional differences in mitochondrial or oxidative activity.

Baseline metabolite–transcript associations were evaluated among participants with matched metabolomic and transcriptomic measurements. Spearman rank correlations were first calculated between each of the 135 baseline metabolites and the oxidative transcript-state score. Partial Spearman correlations were then calculated after adjustment for participant group and sex. Unadjusted and adjusted analyses were treated as separate hypothesis families and corrected independently using the Benjamini–Hochberg false-discovery-rate procedure.

### 2.5. Exploratory Prediction of Acute Acetylcarnitine Response

Prediction analyses included 43 participants with complete paired acetylcarnitine measurements and baseline molecular features. The principal continuous outcome was acute acetylcarnitine response, calculated as post-exercise minus pre-exercise log_2_ relative abundance.

Four baseline metabolites were included in the parsimonious metabolite model: acetylcarnitine, free carnitine, NAD^+^, and NADP^+^. These metabolites were selected before predictive model fitting because they represent complementary components of carnitine-dependent acetyl-group handling and cellular redox metabolism. Their inclusion was not determined using the held-out participant or by maximizing predictive performance.

All prediction procedures were conducted using participant-held-out nested validation. During each outer validation iteration, one participant was completely excluded from model development. Preprocessing, standardization, hyperparameter selection, and model fitting were performed exclusively within the remaining training participants. The held-out participant therefore contributed no information to feature preprocessing or model optimization.

Ridge regression was fitted using standardized predictors. The regularization parameter was selected within the training data from a logarithmic grid ranging from $10^{-4}$ to $10^{4}$ using five-fold inner cross-validation.

TabPFN v2 was implemented using the pretrained tabular foundation-model framework without additional fine-tuning of foundation-model parameters. At each outer validation iteration, only observations from the corresponding training set were supplied to the model before prediction of the held-out participant.

The pathway-informed Transformer was designed as a low-capacity model to limit model complexity relative to cohort size. The architecture contained two Transformer encoder layers, a model dimension of 32, four self-attention heads, a feed-forward dimension of 64, dropout of 0.10, layer normalization, and a linear regression output head. The final network contained 17,697 trainable parameters. Models were optimized using AdamW with an initial learning rate of $1\times 10^{-3}$, weight decay of $1\times 10^{-4}$, batch size of 8, and a maximum of 300 epochs. Early stopping was applied when the inner-validation loss failed to improve for 30 consecutive epochs.

Continuous model performance was evaluated using out-of-fold $R^{2}$, root-mean-square error, and mean absolute error. Uncertainty was quantified using 5000 participant-level bootstrap resamples of the concatenated out-of-fold predictions. Bootstrap 95% confidence intervals were defined by the 2.5th and 97.5th percentiles. Because competing models were evaluated using the same participants, differences in $R^{2}$ were additionally assessed by paired bootstrap resampling.

A secondary binary outcome defined a large acetylcarnitine response as an increase of at least 1 log_2_ unit, corresponding to a minimum twofold increase relative to baseline. This threshold was chosen to represent a readily interpretable large molecular response rather than being selected by maximizing classification performance. Classification performance was summarized using receiver-operating characteristic area under the curve with participant-level bootstrap 95% confidence intervals.

Because prediction of a post–pre change score from its baseline component can be influenced by regression to the mean and mathematical coupling, an additional baseline-adjusted sensitivity analysis was performed. Post-exercise acetylcarnitine abundance was used as the outcome, while baseline acetylcarnitine was included as a mandatory predictor. The incremental predictive contribution of the remaining baseline metabolites was then assessed using the same participant-held-out framework.

### 2.6. Multiple-Testing Strategy

Benjamini–Hochberg FDR correction was applied separately to prespecified and conceptually distinct hypothesis families. The 135 paired metabolite tests within each participant group constituted four independent testing families. The 135 exercise-trained-versus-normally active contrasts of acute metabolite change constituted a separate family. The seven predefined biochemical-class enrichment tests constituted one family. The 135 unadjusted baseline metabolite–oxidative-state associations constituted one family, whereas the corresponding 135 group- and sex-adjusted partial correlations constituted another family. Contextual transcriptomic analyses were corrected independently within each dataset and prespecified contrast. Statistical significance was defined as q<0.05.

Additional muscle and adipose transcriptomic datasets were analyzed within their original paired or cross-sectional study structures and were used to provide biological context rather than external validation of the acute metabolomic findings. Because platforms, tissues, participant characteristics, and preprocessing pipelines differed among datasets, absolute pathway-score values were not compared directly across cohorts.

## 3. Results

### 3.1. Analytical Cohort and Data Completeness

The acute-exercise dataset comprised 47 participants and 93 skeletal-muscle biopsies. Complete paired pre- and post-exercise metabolomics were available for 46 participants: 11 young, 14 normally active older, 16 exercise-trained older, and 5 physically impaired older adults. Matched baseline metabolomic and transcriptomic measurements were available for 45 participants, and 43 participants had complete features for exploratory acetylcarnitine prediction. These analysis-specific denominators were retained rather than restricting every analysis to the smallest subset.

### 3.2. Acute Exercise Produced Distributed Remodeling in Trained Older Muscle

In exercise-trained older adults, 17 of 135 metabolites passed FDR correction. In total, 4 increased and 13 decreased, spanning carnitine handling, central carbon metabolism, amino-acid metabolism, cellular energetics, choline metabolism, and related redox-associated pools. Acetylcarnitine (C2:0) increased by 1.167 $\log_{2}$ units (95% CI 0.422–1.912; q=0.047; approximately 2.25-fold). Glucose increased by 0.745 $\log_{2}$ units (q=0.048), while tryptophan and allantoin increased by 0.414 and 0.417 $\log_{2}$ units, respectively.

**Figure 1:**
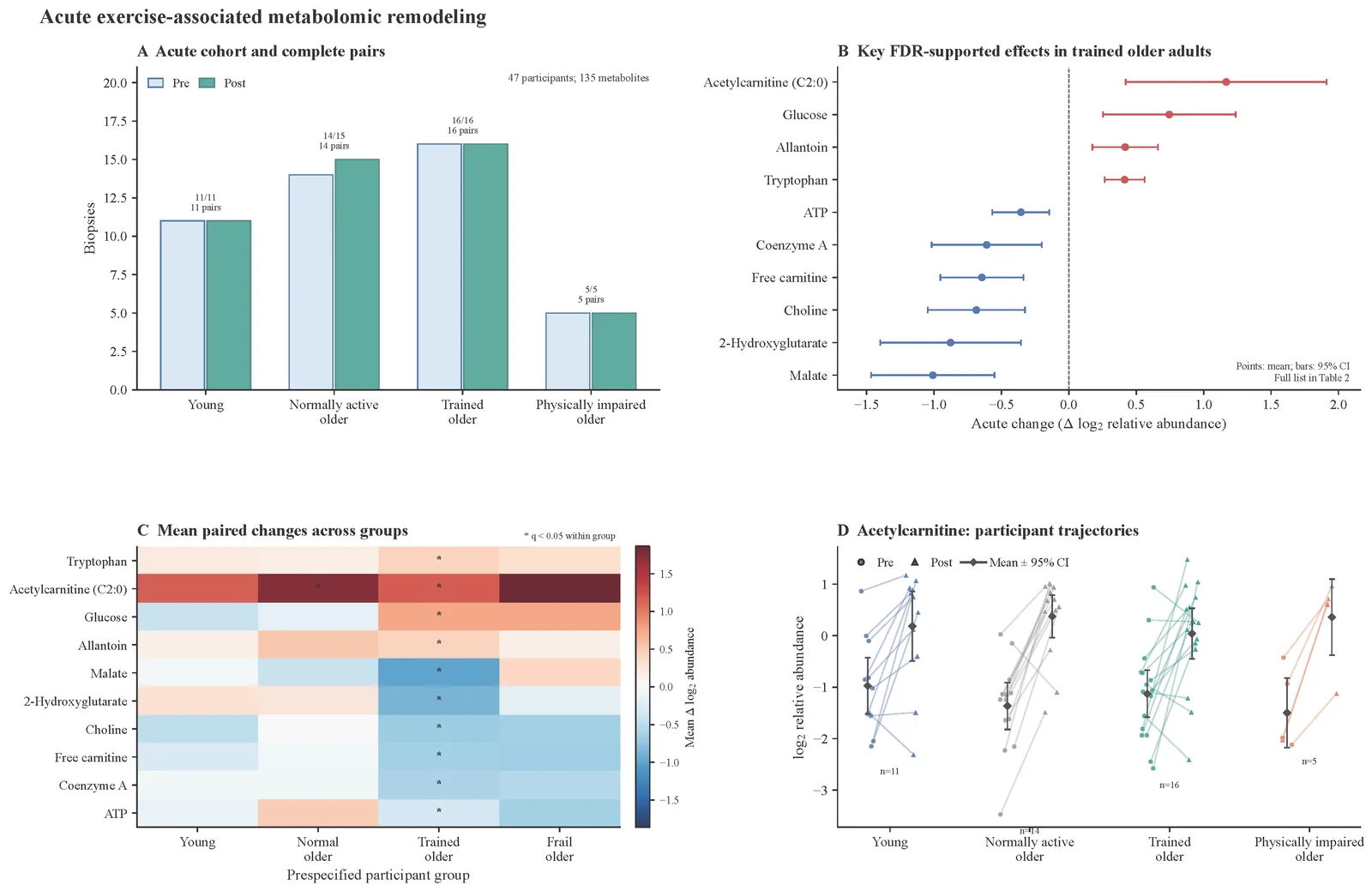
Acute exercise-associated skeletal-muscle metabolomic remodeling. (**A**) Analysis-specific biopsy counts and complete pairs. (**B**) Key FDR-supported effects in exercise-trained older adults; the full 17-feature list is provided in Table 2. (**C**) Mean paired changes for the same key features across prespecified groups; asterisks denote within-group q<0.05. (**D**) Participant-level acetylcarnitine trajectories with group means and 95% confidence intervals. The figure is descriptive of within-group responses and shows the measured acute remodeling pattern.

Malate decreased by 1.008 $\log_{2}$ units (95% CI −1.466 to −0.551; q=0.019), free carnitine by 0.645 (q=0.020), ATP by 0.356 (q=0.036), and choline by 0.686 (q=0.032). Additional FDR-supported decreases were observed for 2-hydroxyglutarate, malonic acid, phosphocreatine, glutamate, glutamine, ornithine, taurine, thymidine monophosphate, and coenzyme A. The reciprocal acetylcarnitine/free-carnitine pattern is compatible with redistribution within the acyl-group pool, but the measured quantities remain pool sizes rather than flux estimates. Key effects and their across-group context are shown in Figure 1; the complete FDR-supported list is provided in Table 2.

Normally active older adults showed three FDR-supported increases: acetylcarnitine (C2:0; Δ$\log_{2}=1.740$, 95% CI 1.177–2.303; $q=2.00\times 10^{-3}$; approximately 3.34-fold), saccharopine (Δ$\log_{2}=1.947$; q=0.015; approximately 3.86-fold), and C3:0 carnitine (Δ$\log_{2}=1.470$; q=0.022; approximately 2.77-fold). No metabolite survived FDR correction in young or physically impaired participants. Direct trained-versus-normally active contrasts of post–pre changes yielded no FDR-supported metabolite. Thus, the broader trained-older within-group profile should not be described as a formally established training-status interaction.

### 3.3. No Predefined Biochemical Class Dominated the Response

The 17 trained-older discoveries were distributed across connected biochemical domains. Enrichment was not significant after correction: carnitine shuttle, 2/9 features, OR 2.11, q=0.737; TCA cycle, 2/9, OR 2.11, q=0.737; amino-acid metabolism, 4/22, OR 1.71, q=0.737; choline/phospholipid, 1/7, q=1.000; glycolysis/carbohydrate, 1/13, q=1.000; NAD/redox, 0/10, q=1.000; nucleotide/energy, 2/27, q=1.000. The complete enrichment results are shown in Table 3.

### 3.4. Baseline Redox-Related Metabolites Were Associated with the Oxidative Transcript State

Baseline metabolite–transcript associations were evaluated in 45 participants with matched measurements. Four metabolites were significantly associated with the oxidative transcript-state score after FDR correction in the unadjusted analysis. The strongest associations were observed for the CoA–glutathione-annotated feature (Spearman ρ=0.570, $q=4.41\times 10^{-3}$), NAD^+^ (ρ=0.559, $q=4.41\times 10^{-3}$), NADP^+^ (ρ=0.518, q=0.012), and 2-hydroxyglutarate (ρ=0.487, q=0.023).

After adjustment for participant group and sex, the associations with the CoA–glutathione-annotated feature and NAD^+^ remained significant. The partial Spearman coefficients were 0.512 for CoA–glutathione (q=0.042) and 0.514 for NAD^+^ (q=0.042). In contrast, the adjusted associations for NADP^+^ (partial ρ=0.454, q=0.124) and 2-hydroxyglutarate (partial ρ=0.412, q=0.146) no longer passed FDR correction.

**Figure 2:**
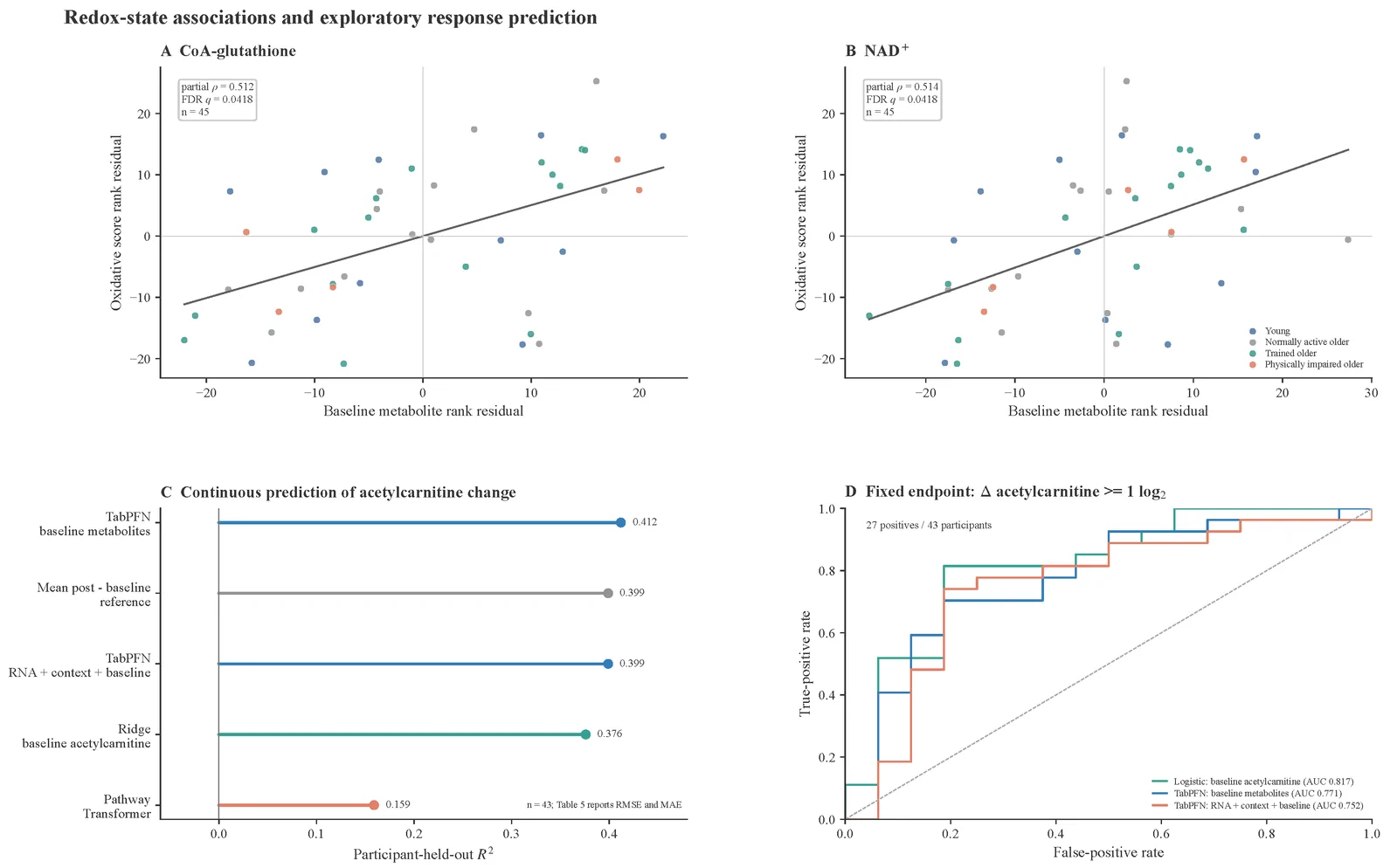
Baseline redox-state associations and exploratory response prediction. (**A**,**B**) Rank-residualized baseline CoA–glutathione and NAD^+^ values plotted against the oxidative transcript-state score after adjustment for group and sex; lines are descriptive least-squares fits, and annotations report the prespecified partial correlations. Points in panels A and B are colored by participant group: young, normally active older, trained older, and physically impaired older. (**C**) Participant-held-out continuous prediction of acute acetylcarnitine change. (**D**) ROC curves for the fixed exploratory endpoint of an increase of at least 1 log_2_ unit.

These findings indicate that selected baseline redox-related metabolites covaried with the transcript-derived oxidative state independently of broad participant-group and sex differences, although the analysis does not establish directionality between metabolite abundance and transcriptomic organization. The complete top associations are shown in Table 4, and the adjusted associations are visualized in Figure 2.

### 3.5. Prediction of Acute Acetylcarnitine Response Was Driven Primarily by Baseline Metabolic State

Prediction analyses included 43 participants with complete baseline molecular features and paired acetylcarnitine measurements. The four-metabolite TabPFN model included baseline acetylcarnitine, free carnitine, NAD^+^, and NADP^+^.

For continuous prediction of post–pre acetylcarnitine change, the four-metabolite TabPFN model yielded an out-of-fold $R^{2}$ of 0.412 (95% CI, 0.079–0.641), RMSE of 0.873, and MAE of 0.686. The mean post-baseline reference yielded $R^{2}=0.399$ (95% CI, 0.054–0.625), RMSE = 0.882, and MAE = 0.690. Ridge regression using baseline acetylcarnitine alone yielded $R^{2}=0.376$ (95% CI, 0.021–0.607), RMSE = 0.899, and MAE = 0.712.

The paired difference in $R^{2}$ between the four-metabolite TabPFN model and the mean post-baseline reference was 0.013 (95% CI, −0.072 to 0.094), indicating little separation between the two approaches. The addition of transcript-derived oxidative features and participant context did not improve continuous predictive performance. The pathway-informed Transformer yielded $R^{2}=0.159$ (95% CI, −0.287 to 0.472), whereas ridge regression using the combined RNA, contextual, and baseline predictor set yielded $R^{2}=-0.066$.

For the exploratory binary endpoint of an acetylcarnitine increase of at least 1 log_2_ unit, baseline acetylcarnitine alone achieved an AUC of 0.817 (95% CI, 0.680–0.941). The four-metabolite TabPFN model yielded an AUC of 0.771 (95% CI, 0.618–0.903), whereas the combined RNA/context model yielded an AUC of 0.752 (95% CI, 0.591–0.887).

The baseline-adjusted sensitivity analysis produced a similar pattern. When post-exercise acetylcarnitine was modeled directly, baseline acetylcarnitine alone yielded an out-of-fold $R^{2}$ of 0.607 (95% CI, 0.364–0.758). Adding free carnitine, NAD^+^, and NADP^+^ increased $R^{2}$ to 0.635 (95% CI, 0.397–0.776). The incremental improvement was modest ($\Delta R^{2}=0.028$; 95% CI, −0.061 to 0.117), indicating that most predictive information was already contained in baseline acetylcarnitine abundance. The continuous benchmarks are summarized in Table 5, and the baseline-adjusted sensitivity analysis is presented in Table 6.

### 3.6. Contextual Transcriptomic Analyses Did Not Replicate the Acute Metabolomic Signal

The longer-term contextual analysis included 24 paired muscle participants and 19 paired adipose participants. No intervention-associated pathway survived correction in either tissue. In older muscle, the oxidative score increased by 0.69 units (95% CI −1.25 to 2.63; paired p=0.453), whereas the younger-group change was 0.99 units (95% CI −0.41 to 2.39; p=0.147). In GSE244120, mean oxidative scores were 84.806 ± 0.591 in metabolically healthy lean participants, 84.870 ± 0.410 in participants with metabolically healthy obesity, and 84.607 ± 0.424 in participants with metabolically unhealthy obesity. The largest contrast, metabolically healthy versus unhealthy obesity, was 0.264 points (95% CI −0.011 to 0.538; p=0.060), and no phenotype contrast survived correction.

## 4. Discussion

### 4.1. Acute Exercise Produced Coordinated but Distributed Remodeling

The principal finding was a broad acute metabolomic response in exercise-trained older muscle. In total, 17 of 135 metabolites passed FDR correction, spanning carnitine handling, central carbon metabolism, energetics, amino-acid metabolism, choline, and related redox-associated pools. This pattern is better described as metabolic remodeling than as selective activation of one pathway. Contraction simultaneously increases ATP turnover, changes substrate demand, perturbs redox conditions, and redistributes acyl groups; an immediate post-exercise biopsy captures the resulting pool-size snapshot [1,2,3]. The direction of carnitine and tryptophan-related remodeling is also consistent with direct human exercise metabolomics in older muscle [28].

The reciprocal increase in acetylcarnitine and decrease in free carnitine is consistent with redistribution within the carnitine/acyl-group pool. Acetylcarnitine formation can buffer acetyl groups when acetyl-CoA generation and utilization are temporarily mismatched. The same direction of acetylcarnitine change in normally active older adults suggests a potentially conserved acute response, while the absence of a corrected trained-versus-normal contrast prevents a training-specific claim. Malate, ATP, phosphocreatine, coenzyme A, glutamate, glutamine, choline, and other features show that the response extended beyond lipid handling.

The null enrichment results are equally informative. Carnitine and TCA classes had an OR of 2.11, but q=0.737, and amino acids had an OR of 1.71, but q=0.737. The correct interpretation is a distributed multi-metabolite response rather than a single dominant biochemical class. Metabolite abundance is not a direct measure of pathway flux, mitochondrial respiration, or clinical benefit.

### 4.2. Training Context Does Not Equal a Formal Interaction

The trained-older group had the largest number of FDR-supported within-group changes, whereas normally active older adults had three. This pattern is compatible with differences in baseline mitochondrial capacity, substrate handling, and response variance, but a difference in the number of discoveries is not itself a statistical interaction. Exercise training can alter metabolic flexibility and muscle molecular state, including in obesity models and in human exercise cohorts, but those contexts do not replace a direct interaction test [29,30,31]. Because no direct trained-versus-normally active contrast survived correction, the data support a broader detectable within-group profile in trained older muscle, not a proven training-dependent amplification.

### 4.3. Cross-Omic Association Defines a Baseline Oxidative State

The cross-omic analysis identified a coherent relationship between selected redox-related metabolites and the transcript-derived oxidative state. The CoA–glutathione-annotated feature and NAD^+^ remained positively associated with the oxidative transcript score after adjustment for participant group and sex. NAD^+^ occupies a central position in oxidative metabolism, electron transfer, and mitochondrial energy production, whereas glutathione-related metabolism contributes to cellular redox buffering and thiol homeostasis. Their concordance with a transcriptional score integrating oxidative phosphorylation, the TCA cycle, fatty-acid metabolism, and branched-chain-amino-acid degradation suggests that metabolite and transcript layers capture partially overlapping dimensions of baseline oxidative-metabolic organization.

The attenuation of the NADP^+^ and 2-hydroxyglutarate associations after adjustment also indicates that not all cross-omic relationships were independent of participant composition. These findings therefore support a focused redox-associated molecular state rather than a globally coordinated metabolome–transcriptome axis. The observed associations characterize baseline molecular organization and should be distinguished from direct measurements of metabolic flux or mitochondrial respiratory capacity.

### 4.4. Baseline State Was More Informative than Complex Response Prediction

The prediction analyses provide a complementary perspective on metabolic plasticity. Baseline metabolite information contained measurable information regarding subsequent acetylcarnitine response, but increasingly complex molecular models did not consistently improve prediction. The four-metabolite TabPFN model produced an out-of-fold $R^{2}$ of 0.412, while its difference from the mean post-baseline reference was 0.013 (95% CI, −0.072 to 0.094). Baseline acetylcarnitine alone produced the highest AUC for the exploratory binary endpoint.

The baseline-adjusted sensitivity analysis further clarified this result. When post-exercise acetylcarnitine was modeled directly with baseline acetylcarnitine included as a mandatory predictor, additional baseline metabolites produced only a modest increase in explained variance ($\Delta R^{2}=0.028$; 95% CI, −0.061 to 0.117). Thus, most predictive information was already contained in baseline acetylcarnitine abundance.

This pattern is biologically plausible. Acute metabolite change reflects both physiological responsiveness and the initial abundance from which the response is measured. Participants with different baseline carnitine pools, redox states, substrate availability, and training histories may therefore arrive at similar post-exercise states through quantitatively different apparent changes. From this perspective, baseline-state characterization may be more robust than attempts to infer a highly individualized acute response from a small multi-omic cohort.

The lack of clear improvement after transcriptomic augmentation is also informative. High-dimensional molecular predictors can increase model flexibility without necessarily contributing independent signal, particularly when the number of participants is limited. The present findings therefore favor parsimonious baseline molecular representations over increasingly complex predictive architectures in this dataset.

### 4.5. Relation to the Original Study

Several broad molecular patterns observed in the present analysis are consistent with those described in the original publication, including acute remodeling of energy-related metabolites, alterations in acylcarnitine-associated metabolism, and heterogeneous responses across fitness groups. Because both analyses are derived from the same underlying cohort, these observations represent consistency within the same cohort rather than independent replication.

The present reanalysis addressed a distinct set of statistical questions. Specifically, we performed metabolite-wise paired testing across the deposited metabolome under explicit FDR control, directly compared acute post–pre responses between exercise-trained and normally active older adults, evaluated biochemical-class enrichment, examined baseline metabolite associations with a prespecified transcript-derived oxidative-state score, and quantified participant-held-out prediction of acetylcarnitine response. The added contribution of the present analysis therefore lies in statistical decomposition of acute metabolic remodeling and evaluation of the boundary between baseline-state association and individualized response prediction.

### 4.6. Strengths and Limitations

Several features strengthen the present analysis, including paired skeletal-muscle biopsies, participant-level matching, explicit FDR control, direct testing of between-group response differences, integration of metabolomic and transcriptomic information, and participant-held-out prediction with simple comparator models.

Several limitations should also be considered. Subgroup sizes were modest, prediction analyses included 43 participants, and no directly comparable external acute-exercise metabolomics cohort was available for validation. Measurements represented relative rather than absolute abundance, and immediate post-exercise sampling did not capture recovery kinetics. Prediction of post–pre change is influenced by baseline acetylcarnitine; the baseline-adjusted analysis therefore tested whether other features contributed beyond the baseline value. Public metadata lacked complete lifestyle, dietary, medication, and physiological covariates.

Sex distribution differed across groups, and reproductive status was unavailable. Sex can influence muscle-fiber composition, mitochondrial capacity, substrate selection, antioxidant and redox buffering, inflammatory signaling, and insulin sensitivity through sex-chromosome effects and gonadal hormones. Estrogen, progesterone, and testosterone vary with menstrual or menopausal status, hormone therapy, contraceptive use, and age-related androgen changes. These factors may alter basal metabolite levels and the acute exercise response. Adjustment for binary sex cannot recover these mechanisms, so the present estimates should not be interpreted as sex-invariant. The deposited 2-hydroxyglutarate feature was not stereoisomer-resolved, and metabolite identities followed the source annotations.

This knowledge gap should be addressed in adequately powered prospective studies with balanced recruitment by sex and reproductive stage. Such studies should record menstrual-cycle phase, menopause status, hormone therapy or contraceptive use, and age-related androgen status. Fasting, exercise workload, sampling time, and biopsy handling should be standardized, with repeated recovery samples. Targeted absolute metabolomics should be paired with muscle transcriptomics or proteomics, fiber typing, mitochondrial-respiration assays, and direct redox measurements. Prespecified sex-by-exercise and reproductive-status interaction analyses, stratified estimates, and external validation would test whether the observed molecular associations are shared or sex-specific.

## 5. Conclusions

Acute exercise produced heterogeneous and distributed remodeling of skeletal-muscle metabolite pools, with prominent changes involving acetylcarnitine, free carnitine, central carbon intermediates, cellular energetics, amino-acid metabolism, and choline-related metabolites. Although exercise-trained older adults exhibited a larger number of FDR-supported within-group changes, direct trained-versus-normally active response contrasts did not survive multiple-testing correction.

At baseline, NAD^+^ and the CoA–glutathione-annotated feature were independently associated with a transcript-derived oxidative state, supporting a redox-associated molecular phenotype spanning metabolite and transcriptional layers. Baseline metabolite information also contained modest predictive information regarding acute acetylcarnitine response, whereas addition of transcriptomic features and more complex architectures produced limited incremental improvement.

Together, these findings suggest that baseline metabolic state may be more reproducibly characterized than individualized acute response magnitude in small multi-omic exercise cohorts. The study provides a framework for integrating paired metabolomic remodeling, cross-omic molecular state, and carefully calibrated predictive analysis in studies of human metabolic plasticity.

## Figures and Tables

**Table 1:** Datasets and analytical roles.

Dataset	Measurement and Role	Samples	Participants/Pairs
MTBLS12824	Acute vastus lateralis metabolomics	135 metabolites; 93 biopsies	47 participants; 46 complete pairs
GSE330697	Matched skeletal-muscle RNA layer	Baseline expression	45 matched participants
GSE158850	Longer-term paired muscle transcriptomics	Muscle array	24 pairs
GSE159809	Longer-term paired adipose transcriptomics	Adipose array	19 pairs
GSE244120	Independent muscle RNA-seq phenotyping	Oxidative pathway scores	50 participants

**Table 2:** FDR-supported acute metabolite changes in exercise-trained older adults.

Metabolite	Δlog_2_	95% CI Lower	95% CI Upper	Fold Change	*p*	*q*
Tryptophan	0.414	0.267	0.562	1.33	$2.50\times 10^{-5}$	0.003
Malate	−1.008	−1.466	−0.551	0.50	$2.86\times 10^{-4}$	0.019
Free carnitine	−0.645	−0.953	−0.337	0.64	$4.54\times 10^{-4}$	0.020
Ornithine	−0.371	−0.569	−0.172	0.77	$1.20\times 10^{-3}$	0.032
Choline	−0.686	−1.047	−0.325	0.62	$1.04\times 10^{-3}$	0.032
2-Hydroxyglutarate	−0.877	−1.399	−0.355	0.54	$2.72\times 10^{-3}$	0.036
ATP	−0.356	−0.567	−0.144	0.78	$2.71\times 10^{-3}$	0.036
Allantoin	0.417	0.174	0.660	1.34	$2.31\times 10^{-3}$	0.036
Taurine	−0.418	−0.669	−0.167	0.75	$2.92\times 10^{-3}$	0.036
Malonic acid	−0.590	−0.942	−0.237	0.66	$2.82\times 10^{-3}$	0.036
Phosphocreatine	−0.455	−0.716	−0.193	0.73	$2.10\times 10^{-3}$	0.036
Acetylcarnitine (C2:0)	1.167	0.422	1.912	2.25	$4.48\times 10^{-3}$	0.047
Glutamate	−0.625	−1.021	−0.229	0.65	$4.28\times 10^{-3}$	0.047
TMP	−1.002	−1.651	−0.354	0.50	$4.92\times 10^{-3}$	0.047
Glutamine	−0.383	−0.635	−0.131	0.77	$5.53\times 10^{-3}$	0.048
Glucose	0.745	0.253	1.237	1.68	$5.63\times 10^{-3}$	0.048
Coenzyme A	−0.609	−1.018	−0.201	0.66	$6.21\times 10^{-3}$	0.049

**Table 3:** Biochemical class enrichment among trained-older discoveries.

Class	Measured	FDR-Supported	OR	*p*	FDR *q*
Amino-acid metabolism	22	4	1.71	0.289	0.737
Carnitine shuttle	9	2	2.11	0.316	0.737
TCA cycle	9	2	2.11	0.316	0.737
Choline/phospholipid	7	1	1.17	0.619	1.000
Glycolysis/carbohydrate	13	1	0.55	0.841	1.000
NAD/redox	10	0	–	1.000	1.000
Nucleotide/energy	27	2	0.50	0.898	1.000

**Table 4:** Baseline metabolite associations with the oxidative transcript-state score.

Metabolite	*n*	Unadj. ρ	Unadj. *p*	Unadj. *q*	Adj. ρ	Adj. *p*	Adj. *q*
CoA–glutathione	45	0.570	$4.42\times 10^{-5}$	$4.41\times 10^{-3}$	0.512	$6.19\times 10^{-4}$	0.042
NAD^+^	45	0.559	$6.53\times 10^{-5}$	$4.41\times 10^{-3}$	0.514	$5.86\times 10^{-4}$	0.042
NADP^+^	45	0.518	$2.65\times 10^{-4}$	0.012	0.454	$2.83\times 10^{-3}$	0.124
2-Hydroxyglutarate	45	0.487	$6.93\times 10^{-4}$	0.023	0.412	0.007	0.146

Metabolite identity follows the annotation provided in the deposited MTBLS12824 dataset.

**Table 5:** Participant-held-out prediction of acute acetylcarnitine change.

Model	Predictor Domain	OOF $R^{2}$	Bootstrap 95% CI	RMSE	MAE
TabPFN v2	Four baseline metabolites	0.412	0.079–0.641	0.873	0.686
Mean post-baseline reference	Baseline reference	0.399	0.054–0.625	0.882	0.690
Ridge regression	Baseline acetylcarnitine	0.376	0.021–0.607	0.899	0.712
TabPFN v2	RNA + context + baseline	0.399	0.047–0.622	0.882	0.689
Ridge regression	Context + baseline	0.276	−0.109–0.543	0.969	0.754
Pathway-informed Transformer	RNA + context + baseline	0.159	−0.287–0.472	1.044	0.812
Transformer	RNA pathway scores + context	−0.040	−0.511–0.329	1.161	0.932
Ridge regression	RNA + context + baseline	−0.066	−0.554–0.308	1.176	0.960

Values represent participant-held-out out-of-fold performance. Confidence intervals were obtained from 5000 participant-level bootstrap resamples.

**Table 6:** Baseline-adjusted sensitivity analysis.

Model	Outcome	OOF $R^{2}$	95% CI	$\Delta R^{2}$ vs. Baseline
Ridge	Post-exercise acetylcarnitine; baseline acetylcarnitine	0.607	0.364–0.758	Reference
TabPFN	Post-exercise acetylcarnitine; four baseline metabolites	0.635	0.397–0.776	0.028
RNA + baseline model	Post-exercise acetylcarnitine	0.616	0.373–0.764	0.0094

## Data Availability

The datasets analyzed in this study are available from the public repositories described in Section 2. Additional processed data, derived analysis files, and analysis code are available from the corresponding author upon reasonable request.
